# A systematic review about long-term results after meniscus repair

**DOI:** 10.1007/s00402-021-03906-z

**Published:** 2021-04-28

**Authors:** Wolf Petersen, Katrin Karpinski, Sebastian Bierke, Ralf Müller Rath, Martin Häner

**Affiliations:** 1 Klinik Für Orthopädie und Unfallchirurgie, Martin Luther Krankenhaus, Berlin, Grunewald, Caspar Theyss Strasse 27-31, 14193 Berlin, Germany; 2 Orthopädische Gemeinschaftspraxis Neuss (OPN), Neuss, Germany

**Keywords:** Meniscus repair, All-inside suture, Inside-out suture, Osteoarthritis, Meniscus suture anchors, Anterior cruciate ligament

## Abstract

**Purpose:**

Aim of this systematic review was to analyze long-term results after meniscus refixation.

**Methods:**

A systematic literature search was carried out in various databases on studies on long-term results after meniscus refixation with a minimum follow-up of 7 years. Primary outcome criterion was the failure rate. Secondary outcome criteria were radiological signs of osteoarthritis (OA) and clinical scores.

**Results:**

A total of 12 retrospective case series (level 4 evidence) were identified that reported about failure rates of more than 7 years follow-up. There was no statistical difference in the failure rates between open repair, arthroscopic inside-out with posterior incisions and arthroscopic all-inside repair with flexible non-resorbable implants. In long-term studies that examined meniscal repair in children and adolescents, failure rates were significantly higher than in studies that examined adults. Six studies have shown minor radiological degenerative changes that differ little from the opposite side. The reported clinical scores at follow-up were good to very good.

**Conclusion:**

This systematic review demonstrates that good long-term outcomes can be obtained in patients after isolated meniscal repair and in combination with ACL reconstruction. With regard to the chondroprotective effect of meniscus repair, the long-term failure rate is acceptable.

**Level of evidence:**

IV.

## Introduction

As a movable articular surface, the menisci play an important role in the load distribution in the knee joint [26]. Other functions of the menisci include stabilization and lubrification of the knee joint [27]. Various studies have shown that the removal of meniscal tissue leads to osteoarthritis in the long-term [6, 7, 34]. However, it could be shown that the progress of post-traumatic osteoarthritis can be slowed down by a meniscus refixation [13, 24, 34]. Therefore, the goal of meniscus surgery is to preserve as much tissue as possible [32].

Meniscal repair is a procedure for which various surgical techniques have been described: open and arthroscopic procedures, inside-out sutures, outside-in sutures, and all-inside sutures [27, 32]. Various factors have been identified that influence the success of meniscal repair (joint stability, associated ACL reconstruction, age, tear shape, methods of stimulating healing) [16, 32]. Various systematic reviews and meta-analyzes report good short- and medium-term results after these suturing procedures with re-rupture rates between 10 and 19% [9, 15, 35].

Follow-up periods between 1 and 3 years count as short-term results, follow-up periods between 4 and 6 years count as medium-term results. When evaluating surgical procedures in orthopedics, however, long-term experience > 7 years is always asked.

Some long-term results after meniscal suturing have already been published, but a systematic review of these studies is lacking. In the past, these studies mainly concerned open procedures or inside-out sutures [4, 5, 10, 14, 21, 23, 30, 33]. Inside-out sutures are usually combined with an additional posteromedial or posterolateral incision, as iatrogenic nerve and vascular injuries can occur in these regions [9, 27, 32]. These additional incisions can be avoided with the use of meniscus suture implants [27]. The first implant generation comprised rigid meniscal fixation implants [8, 9, 15, 17]. The early clinical results were encouraging, but complications such as cartilage injury due to the prominent heads of the implants or implant migration have been described [8, 17]. Therefore, these implants were soon replaced by flexible suture anchor systems [32].

Comparative studies have shown that the early results of these new suture anchor systems are comparable to conventional suture techniques in terms of failure rates [9]. Nerve irritation is even less common when using flexible suture anchors compared to inside-out sutures [9]. So far, however, there has been no comparison of long-term data on flexible suture anchors and traditional meniscus sutures. This is probably because there are no long-term comparative studies on this issue in the literature. To date, a systematic evaluation or meta-analysis of these long-term studies to compare the outcome of flexible suture anchors which the outcome of traditional meniscus repair techniques has not yet been carried out.

Therefore, it is the aim of this systematic review to analyze long-term studies after meniscus repair with regard to the failure rates.

The hypothesis was that the cumulative failure rate of flexible suture implants does not differ from the failure rate of conventional suture techniques.

## Methods

### Search details

Between July 01, 2020 and August 15, 2020, a systematic literature search was carried out in various databases (PubMed, MEDLINE, EMBASE, Scopus, Google scholar) according to PRISMA criteria to identify work in which the long-term results after meniscus refixation was examined. The present study was registered prospectively (www.crd.york.ac.uk/PROSPERO; no.: CRD42020201144).

The following search terms were used: meniscus refixation or meniscus repair or meniscus suture and long-term outcome or failure rate. If a corresponding study was found, related articles were searched in PubMed and searched for relevant publications. In addition, the reference section of relevant studies was also checked.

The main search was carried out by two reviewers (KK and WP). Inclusion and exclusion criteria were used for article selection. Inclusion criteria were: (1) open meniscus repair, (2) arthroscopic meniscus repair, (3) minimum of 7 years follow-up and (4) English language. Exclusion criteria were: (1) no failure rate reported, (2) recommendation and guideline papers, (3) previous systematic reviews or previous meta-analysis.

When multiple articles of one clinical trial were available, the trial with the longest follow-up was included.

### Risk of bias (quality) assessment

A quality assessment tool should be used depending on the study type. The PEDRO scale is used for randomized controlled trials. The Newcastle Ottawa scale should be used for cohort studies. For case series, the tool for evaluating the methodological quality of case reports and case series—as proposed by Murat et al.—should be used [22]. Quality assessment was conducted by WP and KK.

### Data extraction (selection and coding)

After researching the literature according to the specified inclusion and exclusion criteria, WP and KK have extracted the following data from the selected studies:

1. Study details—journal of publication, date of publication, country/countries where the study took place, sample size, study design, inclusion and exclusion criteria.

2. Patient details—age, gender, associated procedures.

3. Outcome measures—failure rate, radiological signs of osteoarthritis, PROMs.

### Outcome measures

Primary outcome measure is the failure rate as reported by the authors. Secondary outcome measures were radiological signs of osteoarthritis as far as reported and various patient reported outcome measures (PROMs) such as Knee Osteoarthritis Outcome Score (KOOS).

### Strategy for data synthesis

WP and KK have constructed a narrative synthesis of the extracted data, structured around the failure rate, radiological signs of osteoarthritis, and PROMS. Tables have been developed to aid the presentation of the extracted data along with the quality assessment. A formal meta-analysis was performed for the primary outcome measure (failure rate). For the cumulative failure rates, number of patients and number of failure rates for three different groups (inside-out, all-inside and hybrid techniques) were summarized and the overall percentages for all three procedures were calculated.

### Statistics

The overall failure rate was calculated using the total number of subjects in the included studies and the number of reported failures. Calculation of 95 confidence intervals was used for the comparison of the calculated failure rates of inside-out, all-inside, and hybrid fixation techniques. The Student’s *t* test was used for the comparison of the calculated failure rates of the different inside-out implants.

## Results

### Search results and study design

**Fig. 1:**
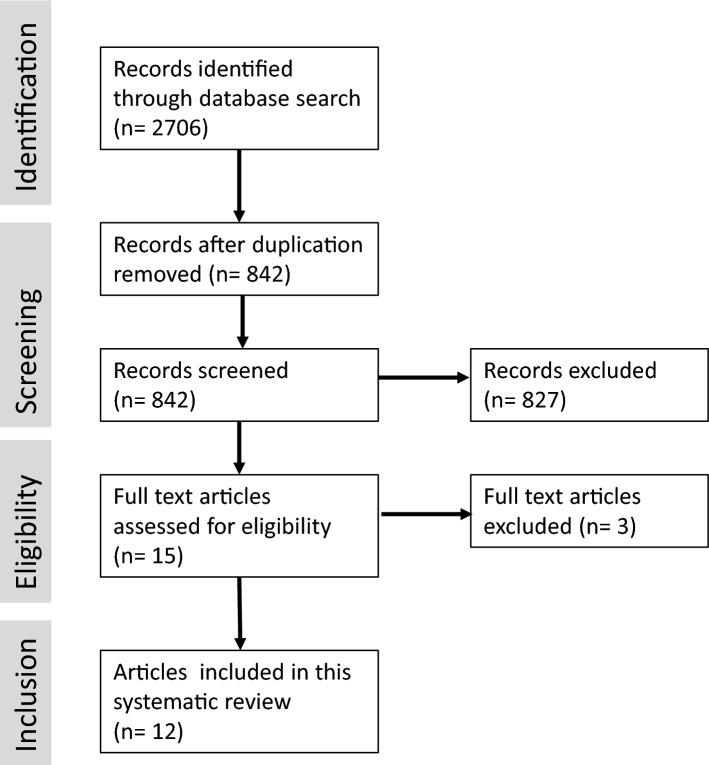
Flowchart showing the search results of the present study

The search results are shown in the Fig. 1. Detailed information about the study designs is provided in Table 1. Out of 15 articles of long-term results about meniscus repair, 3 had to be excluded due to duplicate publications or missing failure rate.

**Table 1:** Design of studies about long-term results after meniscus refixation

Nr.	Author, year	No. of repairs	Mean age of patients (range)	Surgical technique	Study design	Mean follow-up (years)	Isolated (no.)/combined with ACL reconstruction (no.)
1.	Rockborn and Gillquist, 2000 [30]	31	25 (14–43)	Open suture	Retrospective case series	13.5	Isolated repair in stable knees
2.	DeHaven et al. 1995 [5]	33	18.9 (14–27)	Open suture	Retrospective case series	10.8	Isolated stable (8), unstable knee (8), combined with ACL reconstruction (17)
3.	Muellner et al. 1999 [21]	33	35.1	Open suture	Retrospective case series	12.9	Isolated stable (8), unstable knee (3), combined with ACL repair (7)
4.	Johnson et al. 1999 [14]	48	20.2 (14–48)	Arthroscopic inside-out and posterior incision	Retrospective case series	10.9	Isolated meniscus injuries
5.	Melton et al. 2011 [20]	37	28 (20–53)	Arthroscopic inside-out and posterior incision	Retrospective cohort study with control group	10	Combined with ACL reconstruction (44)
6.	Noyes et al. 2011 [23]	33	16.8(10.1–21.9)	Arthroscopic inside-out and posterior incision	Retrospective case series	16.8	Isolated stable (7), combined with ACL reconstruction (26)
7.	Brucker et al. 2011 [4]	26	20.6 (16–25)	Arthroscopic inside-out and posterior incision	Retrospective case series	20.6	Isolated meniscus injuries in stable knees
8.	Steadman et al. 2015 [33]	181	33 (18–70)	Arthroscopic inside-out and posterior incision	Retrospective cohort study with control group	16.1	Isolated stable (40), unstable knee (68), combined with ACL reconstruction (73)
9.	Pujol et al. 2015 [29]	41	22 (9–49)	Arthroscopic all inside refixation	Retrospective case series	11.9	Isolated stable (25), combined with ACL reconstruction (16)
10.	Solheim et al. 2016 [31]	82	33 (14–57)	Arthroscopic all inside refixation	Retrospective case series	10	Stable knees, no concomitant ACL reconstructions
11.	Zimmerer et al. 2018 [37]	63	29 (14–49)	Arthroscopic all inside refixation	Retrospective case series	12.9	Isolated (14), combined with ACL reconstruction (49)
12.	Hagmeijer et al. 2019 [10]	44	16.1 (9.9–18.7)	Arthroscopic all inside refixation, inside-out and hybrid fixation	Retrospective case series	17.6	Isolated

A total of 12 articles were identified that reported about long-term results after meniscus refixation [4, 5, 10, 14, 20, 21, 23, 29–31, 33, 37]. The majority of studies identified were retrospective case series without a control group. In one study, results of meniscus refixation in patients older than 40 years and younger than 40 years was compared [33]. The rest of the studies had no control group.

Three studies were about open meniscus repair [5, 21, 30], five studies were about arthroscopic meniscus repair with inside-out sutures and a posteromedial or posterolateral incision [4, 14, 20, 23, 33], three studies were about the use of all-inside flexible suture anchors [29, 31, 37], and one study was about a hybrid technique using all-inside suture anchors and inside-out sutures [10]. No study examined the failure rate of outside-in sutures.

Table 2 shows the results of the study quality analysis incorporating the tool for evaluating the methodological quality of case reports and case series as proposed by Murat et al. [22]. In this scale, most studies achieved four of four possible points (Table 2). Only for the study by Steadman et al., the full number of points was not awarded [33]. A positive answer to the domain ascertainment was not possible. In this study, 59 of 163 patients who were available for follow-up underwent subsequent arthroscopy [33]. In 15 of these patients, no operative report was available and in 13 patients, a new meniscus tear at the index side was diagnosed, but not counted as failure [33]. This was seen as a high risk of bias with the risk to underestimate the real failure rate in this study.

**Table 2:** Quality assessment with the tool for evaluating the methodological quality of case reports and case series as described by Murat et al. [22]

Nr.	Author, year	Selection	Ascertainment	Causality	Reporting	Overall
1.	Rockborn and Gillquist, 2000 [30]	1	1	1	1	4
2.	DeHaven et al., 1995 [5]	1	1	1	1	4
3.	Muellner et al., 1999 [21]	1	1	1	1	4
4.	Johnson et al., 1999 [14]	1	1	1	1	4
5.	Melton et al. 2011 [20]	1	1	1	1	4
6.	Noyes et al., 2011 [23]	1	1	1	1	4
7.	Brucker et al., 2011 [4]	1	1	1	1	4
8.	Steadman et al., 2015 [33]	1	0	1	1	3
9.	Pujol et al., 2015 [29]	1	1	1	1	4
10.	Solheim et al.,2016 [31]	1	1	1	1	4
11.	Zimmerer et al.,2018 [37]	1	1	1	1	4
12.	Hagmeijer et al., 2019v	1	1	1	1	4

### Primary outcome

Table 3 shows the study results of the failure rates as reported in the different studies which were included in the present systematic review. The failure rates of these studies vary between 5 and 48%.

**Table 3:** Failure rates in long-term studies after meniscus repair

Nr.	Author, year	Failure definition	No. of repairs	Failures	Failure rate (%)
1.	Rockborn and Gillquist, 2000 [30]	Arthroscopic confirmed failure	31	10	29
2.	DeHaven et al. 1995 [5]	Arthroscopic confirmed failure	33	7	21
3.	Muellner et al. 1999 [21]	Arthroscopic confirmed failure	22	2	9
4.	Johnson et al. 1999 [14]	Clinical failure	38	9	24
5.	Melton et al. 2011 [20]	Not stated	22	3	14
6.	Noyes et al. 2011 [23]	Clinical, radiological (MRI) and arthroscopic failure	29	11	38
7.	Brucker et al. 2011 [4]	Arthroscopic confirmed failure	26	6	23
8.	Steadman et al. 2015 [33]	Arthroscopic confirmed failure	163	8	5
9.	Pujol et al. 2015 [29]	Arthroscopic confirmed failure	31	4	13
10.	Solheim et al. 2016 [31]	Arthroscopic confirmed failure	82	32	48
11.	Zimmerer et al. 2018 [37]	Arthroscopic confirmed failure	63	17	27
12.	Hagmeijer et al., 2019 [10]	Arthroscopic confirmed failure	33	14	42

Table 4 summarizes the failure rates for open repair techniques, arthroscopic inside-out, and arthroscopic all-inside. The statistical analysis of the 95% confidence intervals showed that these are almost congruent. Therefore, no significant difference in the failure rates of the different meniscus repair techniques can be assumed.

**Table 4:** Failure rates in long-term studies after meniscus repair

Nr.	Technique	No. of repairs	Failures	Failure rate (%)
1.	Open repair	86	20	23.3
2.	Arthroscopic inside-out with additional posteromedial or posterolateral incision	245	29	25.2
3.	Arthroscopic all inside resorbable and non-resorbable	176	53	30.1
4.	Arthroscopic all inside non-resorbable	94	21	22.3

The study published by Steadman et al. was excluded from the overall failure rate calculation as shown in Table 4 due to the risk of bias for ascertainment (Table 2) [10, 33]. In Steadman’s study, the overall failure rate was only 5%, which is significantly lower than for other studies using the same arthroscopic inside-out technique. Still, due to the high number of patients, this study is very important to mention in this review. Nevertheless, it was excluded when calculating the overall failure rate, as with inclusion of this study, the calculation results may be biased toward a failure rate that is too low. The study by Hagmeijer et al. was also excluded from the overall failure rate calculation because in this study, inside-out sutures were combined with all-inside repair techniques [10].

A closer look at the failure rates of the all-inside procedures used in the papers analyzed shows that the arthroscopic failure rate also depends on the implant. There is a significant difference in failure rates between the Fastfix™ (Smith and Nephew, London, UK) with 22.3% [29, 37] and the Rapidloc™ implant (DePuy Mitek, Raynham, Massachusetts) [31] with 48% failure rate (*P* = 0.043).

### Secondary outcome

Seven studies evaluated radiological outcome regarding signs for osteoarthritis (Table 5). The most frequent radiological osteoarthritis score was the Ahlbäck classification. In all studies, only mild osteoarthritic changes were observed [4, 5, 14, 21, 23, 29, 30]. These changes did not differ much from the uninjured contralateral knee [14].

**Table 5:** Radiological signs for osteoarthritis in long-term studies after meniscus repair

Nr.	Author, year	Radiographic results
1.	Rockborn and Gillquist, 2000 [30]	Ahlbäck grade I: 2 of 31 patients
2.	DeHaven et al. 1995 [5]	Overall: Ahlbäck grade I: 8 of 33 patients Successful repair: Ahlbäck grade I: 4 of 26 patients Failed repair: Ahlbäck grade I: 4 of 7 patients
3.	Muellner et al. 1999 [21]	Fairbank score: 17 patients without degenerative changes, 3 grade 1 and 2 grade 2
4.	Johnson et al. 1999 [14]	35 knees (92%) had no degenerative changes, 3 knees (8%) had minimal degenerative changes On the non-operated knee, 33 knees (97%) had no degenerative findings and 1 knee (3%) had minimal degenerative findings
5.	Melton et al. 2011[20]	Not reported
6.	Noyes et al. 2011 [23]	Radiological IKDC score: normal in 15, nearly normal in 8 and severely abnormal in 3 knees
7.	Brucker et al. 2011 [4]	Ahlbäck grade I: 2, grad II: 2 of 18 patients
8.	Steadman et al. 2015 [33]	Not reported
9.	Pujol et al. 2015 [29]	Ahlbäck grade I: 6, grade II: 2 of 29 patients
10.	Solheim et al. 2016 [31]	Not reported
11.	Zimmerer et al. 2018 [37]	Not reported
12.	Hagmeijer et al. 2019 [10]	Not reported

Ten studies reported different patient reported clinical scores as outcome measures (PROM) such as KOOS, Lysholm or subjective IKDC score [4, 5, 10, 20, 21, 23, 29, 30, 33, 37]. Table 6 summarizes the PROMs of the different studies. The most frequent score was the Lysholm score with mean values between 86 and 98 points. In one study, the mean Lysholm score was 95 points in the repair group and 100 points in the control group [30] and in another study, the Lysholm score did not decrease with time (short-term follow-up vs. long-term follow-up) [29]. In one study, the subjective IKDC score increased significantly from preoperatively to postoperatively [10].

**Table 6:** Clinical scores of long-term studies after meniscus repair

Nr.	Author, year	Failure definition
1.	Rockborn and Gillquist, 2000 [30]	Mean Lysholm score: 95 (89–95)
2.	DeHaven et al. 1995 [5]	Mean Lysholm score: 90 (70–100)
3.	Muellner et al. 1999 [21]	OAK Knee Score: 14 excellent, 6 good and 2 fair or poor
4.	Johnson et al. 1999 [14]	Not reported
5.	Melton et al. 2011 [20]	Mean subjective IKDC score: 84.2 Mean Lysholm score: 98 (20–100)
6.	Noyes et al. 2011 [23]	Cincinatti rating score: 86.5
7.	Brucker et al. 2011 [4]	Mean Lysholm score: 97.8 (85–100) Subjective IKDC: 93% (77–100), Tegner activity scale: 4.2 (3–7)
8.	Steadman et al. 2015 [33]	Lysholm score: < 40y 86 (53–100), > 40 y 86 (53–100) Tegner activity scale: < 40 y 5 (1–9), > 40y 3 (0–7) WOMAC Pain: < 40 y 1 (0–9), > 40 y 2 (0–10); Stiffness: < 40 y 1 (0–5), > 40 y 2 (0–6); Function: < 40 y 3 (0–27), > 40 y 6 (0–31); Total: < 40 y 5 (0–37), > 40 y 10 (0–45) SF-12 Physical component summary: < 40 y 54.0 (25.7–62.0), > 40y 52.3 (33.1–60.3); Mental component summary: < 40 y 53.1 (23.9–61.8), > 40 y 54.6 (36.8–61.8)
9.	Pujol et al. 2015 [29]	Mean Lysholm score: 94.7 (80–100) KOOS: pain 94.3 ± 9; symptoms 90.9 ± 15; ADL 98.7 ± 2; sports 91.1 ± 14; quality of life 91.5 ± 15
10.	Solheim et al. 2016 [31]	Not reported
11.	Zimmerer et al. 2018 [37]	KOOS: pain 91.35; stiffness 86.56; ADL 94.65; sports 80.34; quality of life 77.28 Tegner activity score: 5.57
12.	Hagmeijer et al. 2019 [10]	Mean subjective IKDC score: 92.3 (88.5–100.0) Tegner activity score: 6.53 (5–9)

## Discussion

The results of this systematic review support our initial hypothesis. There is no difference in the reported long term failure rates of meniscus repair using flexible all-inside meniscus anchors or traditional meniscus inside-out sutures. These results support other systematic reviews that compared early- and mid-term results after meniscal repair with flexible all-inside implants and conventional inside-out sutures and could not show any difference between the two methods [9, 15]. However, the long-term failure rates were higher than the cumulative short-term failure rates reported by Grant et al. [9] and Kang et al. [15]. For example, Grant et al. reported long-term failure of 17% for inside-out repairs and 19% for all-inside repairs [9]. This means that meniscal sutures can still fail after initial healing which can be expected after a period of 2 years postoperatively. But it should also be noted that previous systematic reviews of short- and medium-term results also included rigid implants [9, 15]. As stated in the introduction, in clinical practice, these implants no longer matter due to the reported complications [9, 15]. The present systematic review was only able to identify studies that analyzed long-term implants after using flexible meniscal suture anchors. Therefore, the statements made regarding long-term failure rates apply only to flexible all inside meniscus suturing systems. Rigid anchors have largely been abandoned in clinical practice because complications such as cartilage damage from the implant heads or implant migration have been described [8, 17].

With regard to the re-rupture rates after all-inside repair with flexible anchor systems, it should be noted that the reported re-rupture rates differ for individual implants. The mean re-rupture rate for the Fastfix™ implant (Smith and Nephew) was 22.3% [29, 36], whereas the re-rupture rate for the Rapidloc™ implant (DePuy Mitek) was 48% [31]. The difference between the two implants is that the Rapidloc™ is resorbable, while the Fastfix™ is non-resorbable. A biomechanical study has shown that Fastfix™ suture anchors had a significantly higher pull-out strength (94.1 N) than did Rapidloc™ devices (30.3 N) [36]. Another explanation for the difference in the failure rate could be that in the study by Solheim et al., no concomitant ACL reconstructions were included [31]. We therefore believe that it makes sense to compare the two implants separately (Table 4).

With view of these long-term failure rates, the use of flexible all-inside meniscus suture anchors appears to be justified, particularly in the posterior horns of the menisci, because there is a risk of vascular and nerve injuries in these region [27, 32]. An earlier systematic review has shown that the risk of nerve injuries when using conventional inside-out sutures is significantly higher than when using all-inside sutures (9% vs 2%) [9]. The major disadvantage of flexible implants, however, is their high cost and scientific data regarding cost-effectiveness are lacking.

Some of the included studies tried to identify risk factors that affect failure rates after meniscal fixation. Age is a well-known prognostic factor for a meniscal suture [32]. Steadman et al. compared outcome of meniscus repair in patients older and younger than 40 years and could not find any difference in failure rates and outcome scores between these groups [33]. This finding is in accordance with the recently published ESSKA consensus statement that the patient’s age does not appear to affect the failure rate of meniscus repairs of traumatic tears in adults [16]. However, in this systematic review, comparatively high re-rupture rates were identified for young patients between 10 and 22 years of age [10, 23]. In the study by Noyes et al., the re-rupture rate was 38% [23] and in the study by Hagmeijer et al. 42% [10], which is significantly higher than the re-rupture rates found for adults. Both studies indicate that children and adolescents have a higher re-rupture risk than adults. In addition, Hagmeijer et al. reported that the long-term failure rate did not deteriorate compared to the short-term results [10]. This may indicate that the high level of activity of young patients can play a role here.

Another often cited factor influencing the success of a meniscal suture is the stability of the joint with regard to the anterior cruciate ligament [16, 24]. The results of the included studies do not allow a clear conclusion on this question. While DeHaven et al. found a statistical relationship between ACL reconstruction and failure rate [5], the results of the Noyes et al. and Steadman et al. were not significant [23, 33]. Anyhow, it is striking that the studies included in the present systematic review in which the meniscal repair was carried out to a high rate in combination with an ACL reconstruction had lower re-rupture rates for the meniscal repair than studies in which an isolated repair was carried out. It has been speculated not only the stability of the knee but also the surgical procedure (concomitant ACL reconstruction) has a positive effect on meniscus healing [3, 8, 12, 32]. Tunnel drilling should expose the intraarticular space to bone marrow-derived stem cells as well as to growth factors, which may ameliorate the meniscus healing rate [16, 32]. However, in the long-term study by Zimmerer et al., there was no significant difference with regard to the meniscal repair failure rate when comparing groups of simultaneous (11/32) and delayed ACL reconstruction (1/6) [37].

The meniscus preservation also seems to have a positive effect on the result of the anterior cruciate ligament reconstruction [19]. A large (10,000 patients) Scandinavian registration study showed that meniscus resection in combination with ACL reconstruction resulted in poorer clinical results than isolated ACL reconstruction patients or when ACL reconstruction was performed in combination with meniscal repair [28]. The same finding was reported by Melton et al. for the long-term outcome after meniscus repair [20]. In this study, patients with ACL reconstruction and meniscal repair had a mean subjective IKDC of 84.2 compared with a mean score of 70.5 in patients who had undergone ACL reconstruction and meniscectomy [20].

Chronicity also seem to be a prognostic factor for successful meniscus healing. The study by DeHaven et al. found a 33% re-tear rate for chronic repairs as compared with a 14% re-tear rate for acute repairs [5].

The goal of meniscal repair is to prevent osteoarthritis. Comparative groups for patients without meniscal fixation are missing in the studies that could be included in the present systematic review. Therefore, a direct comparison of osteoarthritis rate between meniscus repair and meniscus resection is not possible with the data generated for this systematic review. However, previous studies have shown that the rate of osteoarthritis after meniscus fixation is significantly lower than after partial menisectomy [24, 34]. This observation is supported by the low rates of osteoarthritis described in the long-term studies included in this review.

Some limitations apply for the present systematic review. One limitation is that predominantly case series could be included in the present systematic review. The evidence gained from case series is generally rated as low. The quality of a systematic review always depends on the quality of the studies included. But even if the level of evidence is estimated to be low, case series should not generally be excluded from finding scientific evidence [25]. The incorporation of case series seems to be justified when no other higher level of evidence is available [22]. This frequently applies to long-term results because controlled studies are usually difficult to carry out over a longer period of time. However, since many surgical procedures in orthopedics aim to prevent long-term degenerative changes, long-term experiences are of particular interest in this specialist area. The results of the current systematic review underline this statement, which shows that long-term failure rates worsen compared to the early results. Moreover, a recent Cochrane review compared the reliability of observational studies with that of randomized controlled trials [2]. In this systematic review, the differences were not as significant as previously believed [2]. These authors recommended that more attention should be paid to judging research only on study types. One pitfall of randomized controlled trials, for example, is selection bias because many patients refuse to be included in a controlled trial [25]. Therefore, the results of these trials cannot be transferred to a real-world scenario.

In most of the studies included in this systematic review, failure was defined as need of a surgical revision due to insufficient healing and it is well accepted that the most reliable technique to assess meniscus healing is arthroscopy [32]. However, it is still a subjective examination that depends on the surgeons’ skills. Another option to assess meniscus healing is the magnetic resonance imaging (MRI). Disadvantage of MRI is that signal changes may persist for a long time and changes in MRI may occur even in asymptomatic knees. Partial healing after meniscal sutures can also be asymptomatic [11]. Therefore, we believe that clinical failure assessed by arthroscopy is the most relevant parameter to assess the success of a meniscus suture.

Another disadvantage of this systematic review is that it does not include any studies on outside-in sutures. This is because studies on outside-in sutures did not meet the inclusion criterion “minimum follow-up of 7 years”. Two long-term studies with a minimum follow-up of 2 years and 5 years have also described acceptable failure rates for this refixation technique (12% and 24%, respectively) [1, 18].

## Conclusion

This systematic review shows that long-term results support the clinical practice to repair the menisci whenever possible. No difference in the reported failure rates of meniscus repair using flexible all-inside meniscus anchors or traditional meniscus inside-out sutures was detected. Therefore, the use of both methods—dependent of the localization of the meniscus tear—seems to be justified.
